# Methicillin-resistant *Staphylococcus lugdunensis* in a neonatal intensive care unit: diagnostic challenges and emergence of multidrug-resistance

**DOI:** 10.3389/fmed.2026.1765608

**Published:** 2026-02-19

**Authors:** Marianne Hille Smith, Torunn Gresdal Rønning, Siren Irene Rettedal, Hege Enger, Iren Høyland Löhr, Jon Sundal, Anlaug Vatne, Heidi Syre, Christina Gabrielsen Ås

**Affiliations:** 1 Department of Medical Microbiology, Stavanger University Hospital, Stavanger, Norway; 2 The Norwegian MRSA Reference Laboratory, Department of Medical Microbiology, Clinic of Laboratory Medicine, St. Olav’s Hospital, Trondheim University Hospital, Trondheim, Norway; 3 Department of Clinical and Molecular Medicine, Norwegian University of Science and Technology, Trondheim, Norway; 4 Department of Simulation-Based Learning, Stavanger University Hospital, Stavanger, Norway; 5 Faculty of Health Sciences, University of Stavanger, Stavanger, Norway; 6 Department of Clinical Science, University of Bergen, Bergen, Norway; 7 Department of Medicine, Stavanger University Hospital, Stavanger, Norway; 8 Department of Paediatrics, Stavanger University Hospital, Stavanger, Norway

**Keywords:** coagulase-negative staphylococci, *mecA*, methicillin-resistant *Staphylococcus lugdunensis*, neonatal intensive care unit, outbreak, SCC*mec*, screening

## Abstract

**Background:**

*Staphylococcus lugdunensis* is a species within the group of coagulase-negative staphylococci (CoNS), typically regarded as a commensal organism residing on human skin. However, it has increasingly been implicated in a range of clinically significant infections, including bacteremia, particularly in preterm neonates. Notably, *S. lugdunensis* exhibits sensitivity to a broad spectrum of antibiotics, and methicillin-resistant strains (MRSL) remain uncommon.

**Aim:**

This study aimed to document the identification of methicillin-resistant *S. lugdunensis* in an extremely premature neonate, emphasizing the diagnostic challenges in detecting *mecA*-mediated resistance and characterizing its unusual resistance determinants, while highlighting the implications for outbreak potential in highly vulnerable neonatal intensive care unit populations.

**Methods:**

Clinical data were collected retrospectively from the patient’s electronic medical journal. MRSL screening and identification were performed with chromogenic media and MALDI-TOF, respectively. Antimicrobial susceptibility testing (AST) was performed according to EUCAST methods, and whole genome sequencing was performed using Illumina and Nanopore technology.

**Results:**

*S. lugdunensis* was isolated from nasal sores in an extremely premature neonate. Although initial AST indicated susceptibility to methicillin, a locally introduced area of technical uncertainty prompted further analysis, which led to the detection of *mecA* by PCR. Screening with chromogenic MRSA plates revealed MRSL colonization in the nose, throat and perineum of the neonate. The MRSL strain belonged to sequence type 3 and displayed an unusual AST profile, caused by SCC*mec* and a multidrug-resistance plasmid.

**Conclusion:**

We report a case of MRSL in an extremely premature neonate, which was the index patient in a neonatal intensive care unit outbreak, and highlight the diagnostic challenges faced in detection, screening and AST. Furthermore, we report the unusual antimicrobial susceptibility profile of this MRSL strain, caused by a multidrug-resistance plasmid with potential for transmission among staphylococci.

## Introduction

*Staphylococcus lugdunensis* is a species of coagulase-negative staphylococci (CoNS), considered to be a human skin commensal (1). Colonization is estimated to occur in 30–50% of individuals, mainly in the inguinal area and the nasal cavity (2). However, it has also been documented as the cause of a variety of infections, including skin and soft-tissue infections, bone, joint and prosthetic joint infections, urinary tract infections, peritonitis, bacteraemia, as well as endocarditis (3, 4). While the incidence of *S. lugdunensis* infections is typically reported to be low, 5–53 per 100,000 persons per year (4), these infections are frequently characterized by high virulence, with clinical manifestations that mirror those of *S. aureus* (4).

Unlike most other CoNS, *S. lugdunensis* remains remarkably susceptible to most antibiotics. Among the reasons for this is a closed pangenome, with multiple systems to prevent horizontal gene transfer, meaning that gain or loss of accessory genes is less common (5). However, methicillin-resistant *S. lugdunensis* (MRSL) have been reported, and appear to be restricted to specific clonal backgrounds, including sequence type (ST)3, ST6, ST38, ST44, ST42 and ST27 (1, 6, 7), while described staphylococcal cassette chromosome *mec* (SCC*mec*) types in MRSL include type II, IV and V (6, 7).

The first case report in the literature described an MRSL infection in a premature neonate in Singapore in 2002 (8). Since then, MRSL has been isolated from patients in multiple countries worldwide (9), with the first documented Nordic case from Sweden in 2011 (10). Outbreaks of CoNS, specifically *S. epidermidis*, *S. heamolyticus* and *S. capitis,* have also been documented in neonatal intensive care units (NICUs) (11). However, the capacity of *S. lugdunensis* to spread and cause outbreaks has not been documented. In most countries, there are established screening-methods and infection prevention and control (IPC) measures to prevent spread of methicillin-resistant *Staphylococcus aureus* (MRSA) in healthcare institutions. However, there are no standardized screening and IPC measures to detect and handle colonization with CoNS, including MRSL. Thus, outbreaks of CoNS in hospitals and healthcare institutions might go undetected.

In this study, we report a case of MRSL in an extremely premature neonate admitted to the NICU at Stavanger University Hospital in Norway, which was the index patient in an MRSL outbreak.

## Results

### Case presentation

**Figure 1:**
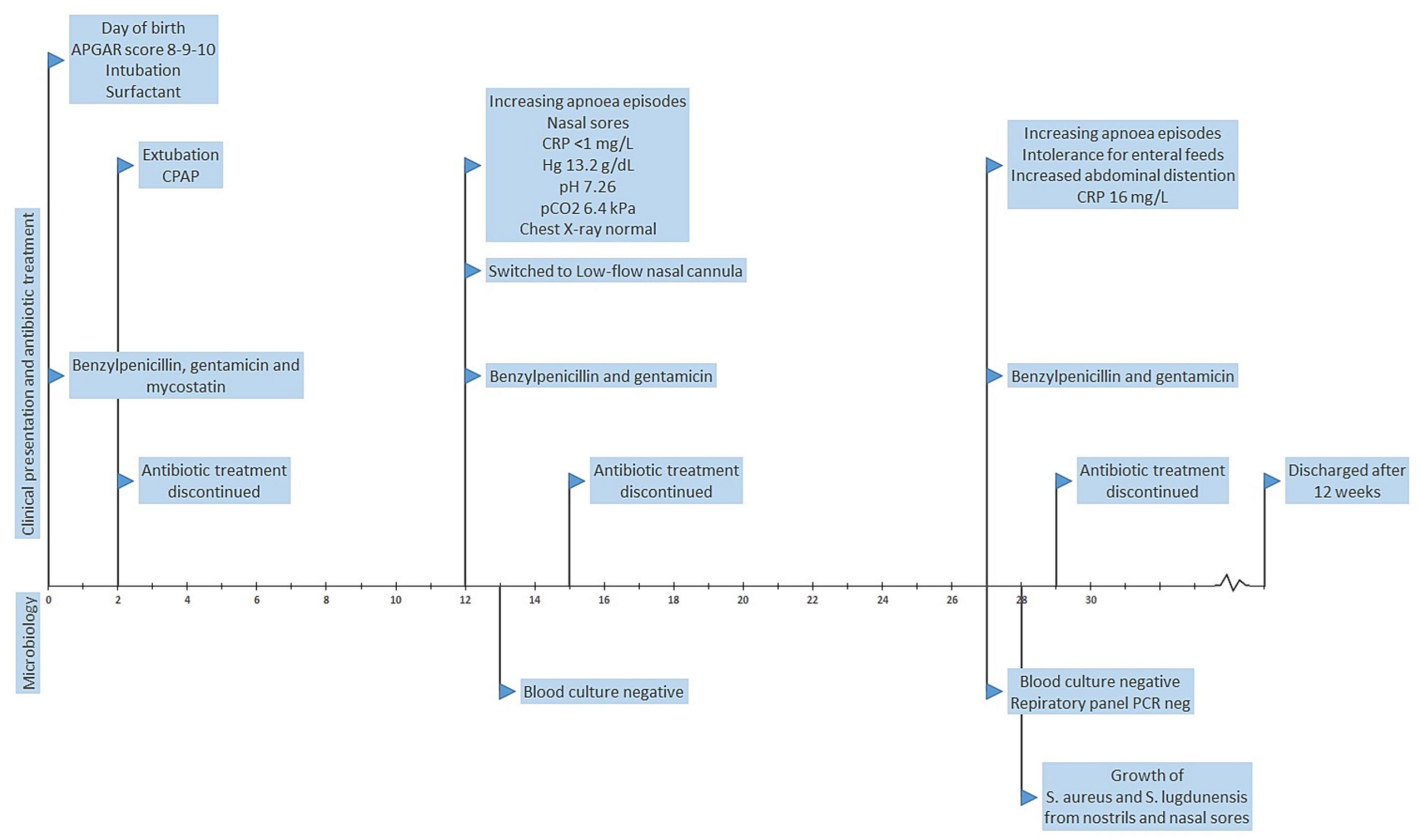
Timeline summarizing the clinical presentation, antibiotic treatment and microbiology of a premature neonate with methicillin-resistant *S. lugdunensis.*

An extremely premature neonate born at gestational age 24 + 2 weeks and days with birth weight 765 g, admitted to NICU at Stavanger University Hospital, Norway November 2020 (Figure 1). The mother was GBS-negative but nonetheless received intrapartum benzylpenicillin at a dose of 1.2 g six times daily from the time of admission, and this regimen was continued for 48 h. The neonate was born after vaginal delivery, and the APGAR score was 8–9–10. Although breathing spontaneously at 1 min, the neonate was intubated shortly after birth for administration of surfactant. Venous and arterial umbilical catheters and a nasogastric tube were inserted. First line empirical antibiotics benzylpenicillin and gentamicin were administered but discontinued after 48 h. In line with national guidelines, the neonate received antifungal prophylaxis (mycostatin). The neonate was extubated day 2 to continuous positive airway pressure (CPAP). Echocardiography revealed a small patent foramen ovale, otherwise the cardiological examination was normal.

On day 12, the neonate presented with increasing apnoea episodes, and small sores with secretion were observed in both nostrils. The level of C-reactive protein (CRP) was <1 mg/L, hemoglobin 13.2 g/dL, pH 7.26 and pCO_2_ 6.4 kPa. Chest X-ray was normal. Blood cultures were negative. Benzylpenicillin and gentamicin were administered on suspicion of sepsis but discontinued after 48 h. CPAP was discontinued in favor of low-flow nasal cannula day 12.

On day 27, the neonate again suffered from increasing apnoea episodes, in addition to intolerance for enteral feeds and increased abdominal distention. CRP was slightly elevated at 16 mg/L. Benzylpenicillin and gentamicin were for the third time administered for 2 days before being discontinued. PCR of nasopharynx secretion (FilmArray, BioFire® Respiratory Panel 2.1 plus) was negative. Blood cultures were negative. However, swabs from nostrils and nasal sores showed growth of *S. lugdunensis* and *S. aureus* 1 day later. The neonate improved clinically without need for further antibiotic treatment. The neonate was discharged from the hospital 12 weeks after birth.

### Microbiological detection and antimicrobial susceptibility testing

Cultivation of swabs from the nasal sores revealed growth of *S. lugdunensis* (MALDI-TOF score 2.26) and *S. aureus*. AST revealed a *S. aureus* with no phenotypic resistance to tested antibiotics. AST of *S. lugdunensis* using cefoxitin disk diffusion yielded a zone diameter of 25 mm. With the cefoxitin breakpoint at the time of testing, this would be interpreted as susceptible. However, due to the locally introduced ATU, the strain was selected for *mecA/C*-PCR, which confirmed the presence of *mecA*.

**Figure 2:**
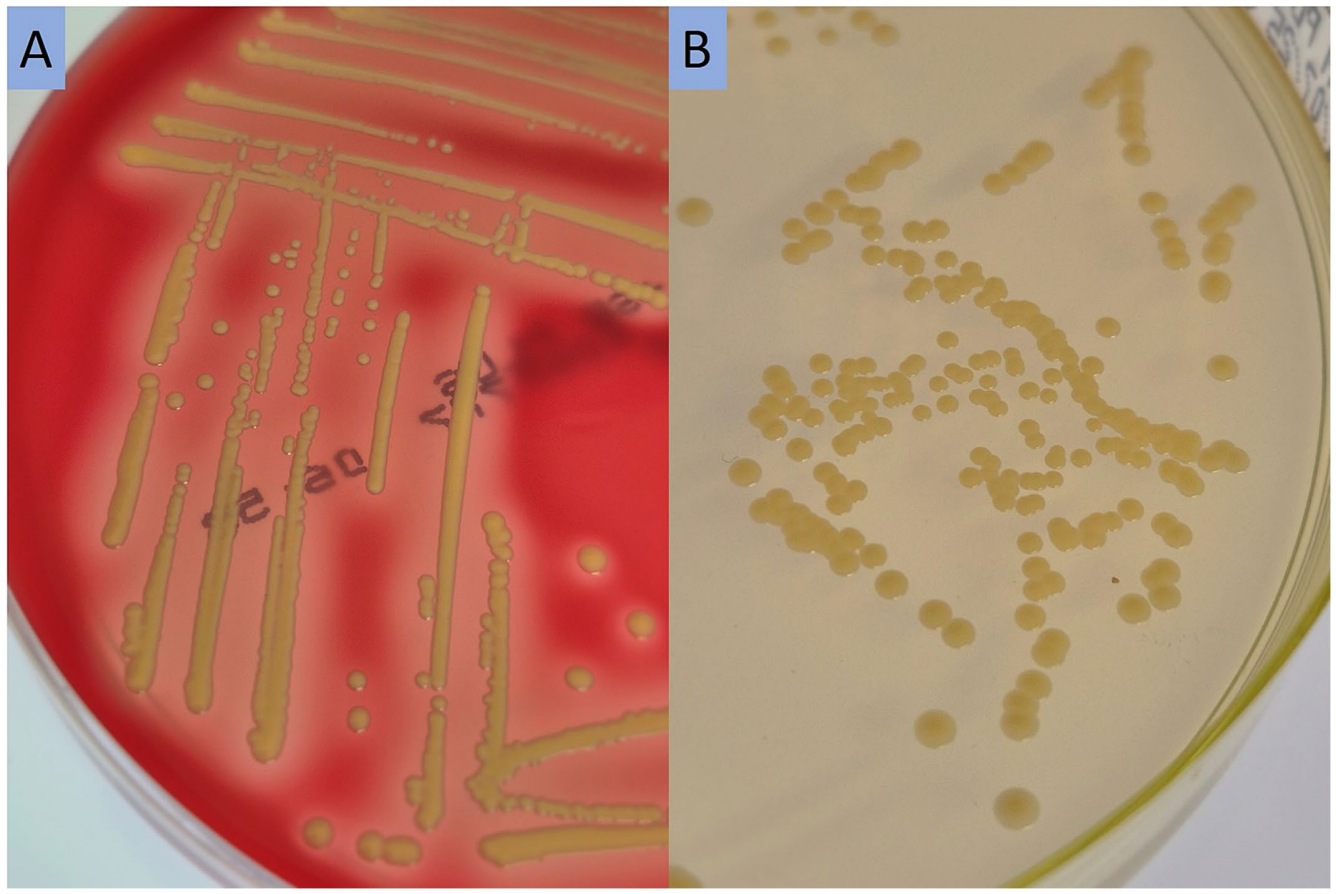
Growth of methicillin-resistant *S. lugdunensis* on sheep blood agar **(A)** and CHROMAgar™ MRSA **(B)**, incubated at 35 °C for 48 h.

Although the CHROMAgar™ MRSA plates are not specifically validated for the detection of MRSL, the MRSL isolates from screening samples grew on the plate in this case, and the additional blood agar supplemented with antibiotics was not necessary for detection. *S. lugdunensis* grew with slightly yellow, shiny colonies with β-hemolysis on blood agar, and with similar colonies on CHROMAgar™ MRSA (Figure 2).

Further susceptibility-testing of the *S. lugdunensis* strain revealed a cefoxitin minimum inhibitory concentration (MIC) of 32 mg/L (resistant > 4 mg/L). By disc diffusion, the strain was furthermore resistant to gentamicin and tetracycline, susceptible with increased exposure (I) to ciprofloxacin, and susceptible to the remaining antibiotics tested (Table 1).

**Table 1:** Results from antimicrobial susceptibility testing by disk diffusion of the methicillin- resistant *S. lugdunensis* strain.

Antibiotic	Zone (mm)	MIC (mg/L)	Interpretation	Comment
Cefoxitin	25		S	Within local ATU
Cefoxitin MIC		32	R	
Gentamicin	16		R	
Tetracycline	11		R	
Ciprofloxacin	34		I	
Erythromycin	32		S	
Clindamycin	32		S	
Fusidic acid	34		S	
Trimethoprim-sulfamethoxazole	38		S	
Linezolid	32		S	
Rifampicin	45		S	
Mupirocin	45		S	
Vancomycin MIC		1	S	
Daptomycin MIC		0.5	S	

Interpreted according to EUCAST breakpoint tables version 11.0 (20).

### Infection control measures and screening methods

After the detection of MRSL in the clinical sample, the hospital infection control team was consulted. The patient and her parents were isolated in a separate room with contact isolation and were screened for MRSL colonization by the same local protocol as for MRSA. Screening of the neonate revealed colonization of MRSL in the nose, throat and perineum, while her parents were screening negative. However, 2 months later, the mother was diagnosed with mastitis by her general practitioner, and MRSL was subsequently detected in a breast milk sample. At 6 months follow-up, the girl and her parents were MRSL negative, and no further screening was performed.

Detection of MRSL in the index patient prompted screening of other infants in the NICU, their parents, and clinical personnel using CHROMAgar™ MRSA plates followed by MALDI-TOF identification. As reported previously, this revealed that four patients and three staff members were colonized. Furthermore, two patients developed sepsis, one of whom had MRSL in blood culture (12).

### Molecular characterization of the MRSL strain

**Figure 3:**
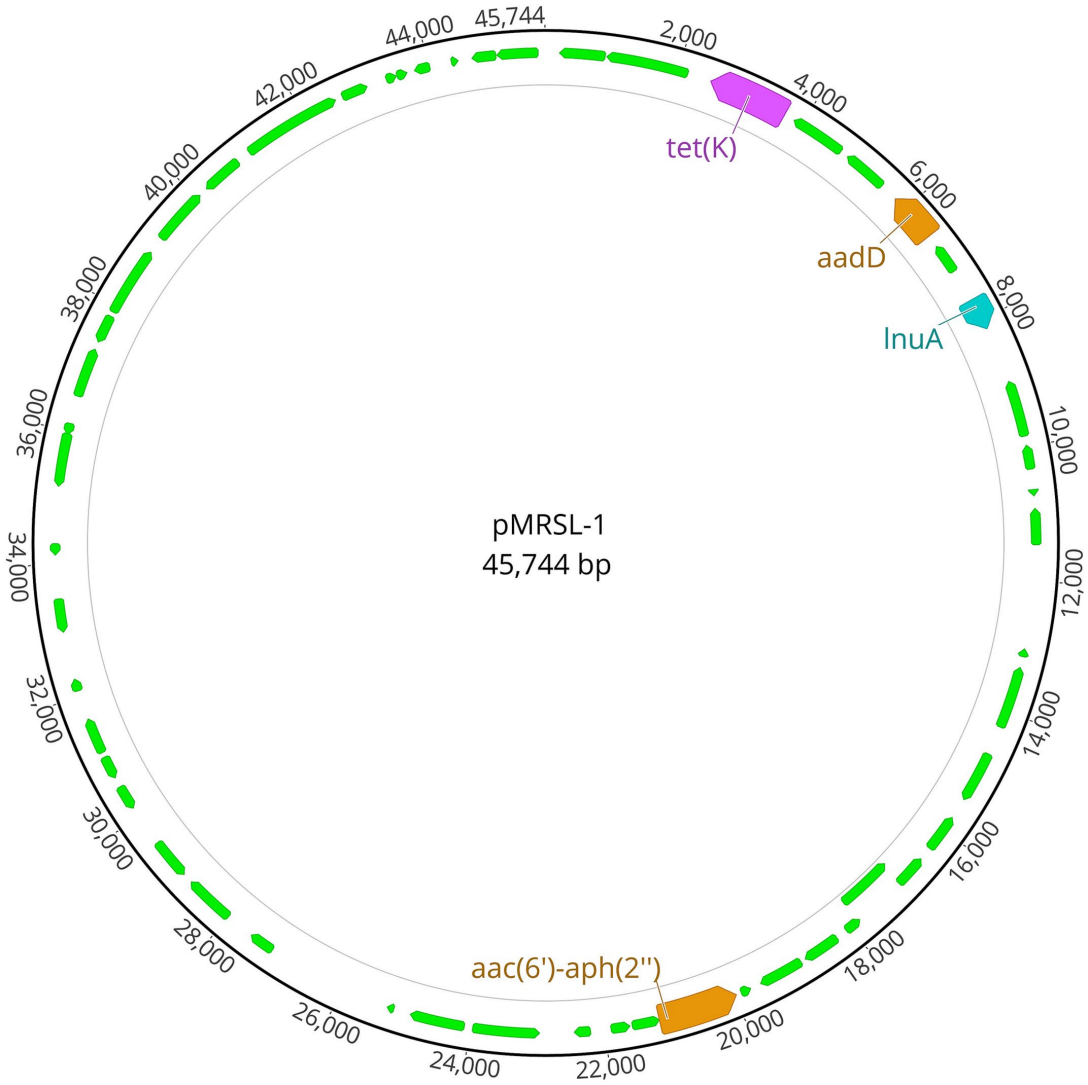
Detected multidrug-resistance plasmid pMRSL-1. Illustration created with Geneious Prime.

The MRSL strain isolated from the premature neonate was whole genome sequenced, yielding a 2,594,971 bp chromosome and a 45,744 bp plasmid named pMRSL-1 (Figure 3). The genome has a total GC content of 33.7% and encodes 2,752 protein-coding genes and 5 rRNA operons. *In-silico* typing revealed that the strain belonged to ST3 and had a chromosomal class IV (2B) SCC*mec* with *mec*A and chromosomal *blaZ*. Other genes conferring antibiotic resistance were plasmid-located, and included *aadD*, *tet(K)*, *lnuA* and a truncated, yet functional *aac(6′)-aph(2″)-gene*. The plasmid was potentially mobilizable (class MOB_V_) and belonged to plasmid taxonomic unit (PTU) Bac21. It had a grade II host-range (13), which suggests a narrow host distribution, and closely related plasmids (mash distance ≥0.99 and *p*-value <0.01) were primarily found in other staphylococci, mainly *S. aureus* (43.8%) and *S. epidermidis* (15.6%). Although similar multidrug-resistance plasmids have been characterized in *S. lugdunensis* previously (14), the best hit using nBLAST covered only 64% of the plasmid.

There were detected 34 virulence-associated genes in the MRSL strain, including type 8 capsular polysaccharide genes, a type VII secretion system and iron acquisition system, as well as a potential sphingomyelinase-c toxin (Supplementary Table S1). Comparative analysis with the functional lugdunin operon of *S. lugdunensis* N920143 (15) demonstrated that *lugA*, *lugB*, and *lugC* in the MRSL strain each harbor multiple frameshift mutations that introduce internal stop codons. These disruptions indicate that the lugdunin biosynthetic operon is degenerated and unlikely to encode a functional antimicrobial product.

## Discussion

In this study, we describe a case of MRSL in an extremely premature neonate admitted to a NICU at Stavanger University Hospital in Norway. Neonates admitted to the NICU, and particularly those born extremely preterm, are at high risk of developing infections because of their immature immune system, prolonged hospitalization, and frequent use of invasive devices and antibiotics (16). The neonate presented with recurrent apnoea episodes and nasal sores and underwent three courses of empirical antibiotic therapy due to clinical signs of sepsis. However, blood cultures were consistently negative. MRSL was isolated from nasal sore secretions on day 27 and later detected in screening samples from the nose, throat, and perineum. Although clinical signs suggestive of infection were observed, *S. aureus* was concurrently isolated from nasal secretions, complicating the attribution of pathogenicity to MRSL. Thus, it remains unclear whether MRSL was the causative agent of infection or merely a transient colonizer in this case. The neonate’s clinical condition improved without need for targeted antimicrobial therapy, and no further signs of systemic infection developed. Nevertheless, MRSL colonization likely led to nosocomial transmission within the NICU, and the patient was identified as the index case in an MRSL outbreak, previously reported by Dahl et al. (12). To our knowledge, this was the first reported outbreak of MRSL in the literature, highlighting the potential for *S. lugdunensis* to spread within healthcare settings. The source of the MRSL strain in this case is unknown, and the index patient’s parents had negative screening samples during the hospitalization. Since *S. lugdunensis* is most commonly found in areas with excessive apocrine glands (4), MRSL colonization at other sites cannot be excluded. This raises the question whether MRSL screening should also include additional potential colonization sites. However, transmission of MRSL may have occurred from colonized staff or from a common environmental source not sampled during the outbreak.

During the MRSL outbreak, two neonates developed sepsis, one of whom had growth of the MRSL outbreak strain in blood culture (12). Thus, it is clear that the MRSL strain did have potential for causing invasive infection in these vulnerable patients. Previous studies have also reported on the infective potential of *S. lugdunensis* in this patient population, with extremely preterm NICU patients developing sepsis with *S. lugdunensis* (8, 17). The incidence of *S. lugdunensis* infections has likely been underreported before MALDI-TOF became standard practice in clinical microbiological laboratories. Unlike many other CoNS, *S. lugdunensis* appears to primarily constitute a true pathogen rather than a contaminant or colonizing organism when detected in clinical samples (18).

AST of CoNS can be challenging, and several methodologies have been evaluated to find an accurate method for the detection of methicillin resistance. A previous study by Cheng-Yen Kao et al. (19) showed that cefoxitin disk diffusion was less sensitive to detect MRSL than oxacillin agar dilution, suggesting that cefoxitin disk diffusion might miss some MRSL strains with oxacillin MIC values close to the breakpoint. Disk diffusion is however used routinely in most clinical laboratories in Norway, including ours, in addition to *mecA*-detection. According to EUCAST guidelines (20) at the time of detection, the MRSL strain with zone diameter of 25 mm should have been reported as cefoxitin susceptible. However, due to the locally defined ATU, *S. lugdunensis* was interpreted as potentially methicillin-resistant, which was confirmed by *mecA* PCR. This further illustrates the challenges with detection of MRSL. Cefoxitin breakpoints for *S. lugdunensis* were, however, revised in January 2022 (sensitive ≥ 27 mm, resistant < 27 mm, ATU 27 mm and MIC > 4 mg/L) (21) for a more sensitive detection of methicillin-resistance, which will most likely increase the detection rate of MRSL in the future.

*S. lugdunensis* has often been described as unexpectedly susceptible to most widely used antibiotics, with resistance to beta-lactams, macrolides and aminoglycosides being particularly uncommon (1). The prevalence of methicillin-resistance in *S. lugdunensis* ranges from 0 to 8.3% (22, 23), with the exception of Taiwan, were the prevalence was 21% due to an endemic ST6 clone (24). Recent data from Norway also show low levels of antimicrobial resistance for most antibiotics tested, including no detection of MRSL in 2023 (25). The MRSL strain described in this study belonged to ST3 and had SCC*mec* type IV (2B), as previously described (6). WGS also uncovered a large plasmid harboring multiple antibiotic resistance genes, encoding resistance to aminoglycosides, tetracycline and lincomycin, suggesting a role in transmission of multi-drug resistance. This plasmid likely originated from another staphylococcal species, given its similarity to those found in *S. aureus* and *S. epidermidis*, and its presence in this MRSL strain suggests a significant potential for transmission of resistance traits into *S. lugdunensis*, posing a future public health threat.

## Conclusion

In this study we report a case of methicillin-resistant *S. lugdunensis* in an extremely premature neonate which was the index patient in a NICU outbreak, highlighting the infective and outbreak potential of MRSL in healthcare settings. We furthermore describe the diagnostic challenges of detection, screening and AST of MRSL, as well as molecular characteristics of the MRSL strain, emphasizing the detection of a multidrug-resistance plasmid with transmission potential.

## Materials and methods

### Clinical data

Clinical data, including gestational age, birth weight, treatment and complications during NICU stay, was collected retrospectively from the patient’s electronic medical journal.

### Screening and identification

Screening samples of the patient and parents from nose, throat and perineum were obtained and placed in Amies transport medium (ESwab®; Copan). The samples were cultivated on chromogenic agar supplemented with methicillin (CHROMAgar™ MRSA; CHROMAgar) routinely used for MRSA-screening. MRSA displays pink to mauve colonies on this agar, while other bacteria may have blue, white or slightly yellow colonies. Screening samples were simultaneously cultivated on sheep blood agar supplemented with colistin and aztreonam for selection of Gram-positive bacteria. Agar plates were incubated at 35 °C for 48 h, and growth evaluated every 24 h. Suspected *S. lugdunensis* colonies were identified by MALDI-TOF (Bruker Daltonics). Confirmed *S. lugdunensis* strains were subjected to PCR (GeneXpert® MRSA NxG; Cepheid) for *mec*A/C detection.

### Antimicrobial susceptibility testing

Antimicrobial susceptibility testing (AST) was performed according to the European committee on antimicrobial susceptibility testing (EUCAST) (26) on Mueller Hinton agar (OXOID). Screening for methicillin-resistance was performed with cefoxitin disk diffusion (30 μg, OXOID). Further susceptibility-testing was performed with agar gradient diffusion methodology (MIC Test Strip, Liofilchem).

The EUCAST cefoxitin breakpoint for *S. aureus* and CoNS (other than *S. epidermidis*) was 22 mm (sensitive ≥ 22 mm, resistant < 22 mm) and the epidemiologic cut-off value (ECOFF) 22 mm at the time of testing (20). For CoNS not identified to species level, a zone diameter of ≥25 mm was considered susceptible and <22 mm resistant. In our laboratory, a defined area of technical uncertainty (ATU) was applied during antimicrobial susceptibility testing (AST) of *S. aureus*, *S. lugdunensis*, and *S. argenteus* against cefoxitin (30 μg). Isolates exhibiting inhibition zones between 22 and 26 mm were subjected to *mecA/C* PCR analysis when deemed clinically relevant. The adjusted ATU was introduced after repeated observations of unusually large cefoxitin inhibition zones in our quality-control strains, raising concerns that the standard criteria might underestimate resistance. This modification was therefore implemented to reduce the risk of false-susceptible results in routine screening.

### Whole genome sequencing

Whole genome sequencing (WGS) was performed at the Norwegian MRSA reference laboratory, St. Olav’s hospital, Trondheim University hospital. Briefly, isolation of DNA was performed with an EZ1 Advanced XL System (Qiagen), while WGS was performed on a MiSeq system, using the Nextera XT DNA sample preparation kit and MiSeq® Reagent Kit v3 (600 cycle; Illumina). The strain was also sequenced using the rapid sequencing kit (SQK-RAD004) with a flongle flow cell (FLO-FLG001) on a MinION instrument (Oxford Nanopore Technologies). The nanopore data were assembled using Flye assembler 2.7 (27), and polished with Illumina-data using Racon 1.4.20 (28). *In silico* MLST was performed using mlst 2.19.0 (28) and SCC*mec* typing using SCC*mec*Finder 1.2 (29). Acquired resistance genes were predicted using ResFinder 4.7.2 (30). Plasmid classification and typing were performed using COPLA (31) and PLSDB (32). Virulence genes were detected using abricate (33) against the VirulenceFactor DataBase (VFDB) (34) with identity ≥60% and coverage ≥50%.

## Data Availability

The datasets presented in this study can be found in online repositories. The names of the repository/repositories and accession number(s) can be found at: https://www.ncbi.nlm.nih.gov/genbank/, PRJNA866316.
